# Systematic Analysis of Monoterpenes: Advances and Challenges in the Treatment of Peptic Ulcer Diseases

**DOI:** 10.3390/biom10020265

**Published:** 2020-02-10

**Authors:** Larissa Lucena Périco, Maycon Tavares Emílio-Silva, Rie Ohara, Vinícius Peixoto Rodrigues, Gabriela Bueno, José Maria Barbosa-Filho, Lúcia Regina Machado da Rocha, Leônia Maria Batista, Clélia Akiko Hiruma-Lima

**Affiliations:** 1 Department of Physiology, São Paulo State University (Unesp), Institute of Biosciences, Botucatu, São Paulo 18618-970, Brazil; larissalucenaperico@gmail.com (L.L.P.); maycon.silva@unesp.br (M.T.E.-S.); rieohara@gmail.com (R.O.); viniciuspr42@gmail.com (V.P.R.); gabriela.sbueno17@gmail.com (G.B.); lucia.rocha@unesp.br (L.R.M.d.R.); 2 Department of Pharmaceutical Sciences, Health Sciences Center, Federal University of Paraíba, João Pessoa, Paraíba 58051-900, Brazil; jbarbosa@ltf.ufpb.br (J.M.B.-F.); leoniabatista1@gmail.com (L.M.B.)

**Keywords:** Peptic ulcer disease, monoterpenes, anti-ulcer agent, *Helicobacter pylori*

## Abstract

Peptic ulcer disease (PUD) is a multifactorial and complex disease caused by an imbalance of protective and aggressive factors (endogenous and exogenous). Despite advances in recent years, it is still responsible for substantial mortality and triggering clinical problems. Over the last decades, the understanding of PUD has changed a lot with the discovery of *Helicobacter pylori* infection. However, this disease continues to be a challenge due to side-effects, incidence of relapse from use of various anti-ulcer medicines, and the rapid appearance of antimicrobial resistance with current *H. pylori* therapies. Consequently, there is the need to identify more effective and safe anti-ulcer agents. The search for new therapies with natural products is a viable alternative and has been encouraged. The literature reports the importance of monoterpenes based on the extensive pharmacological action of this class, including wound healing and anti-ulcerogenic agents. In the present study, 20 monoterpenes with anti-ulcerogenic properties were evaluated by assessing recent in vitro and in vivo studies. Here, we review the anti-ulcer effects of monoterpenes against ulcerogenic factors such as ethanol, nonsteroidal anti-inflammatory drugs (NSAIDs), and *Helicobacter pylori,* highlighting challenges in the field.

## 1. Introduction

The term peptic ulcer disease (PUD) is described by the rupture of the defensive barrier of the epithelial mucosa of the stomach and duodenum and is characterized by their inflammatory process and ulcer formation [1]. The ulcers range from superficial epithelial damage to deeper erosions, causing organ bleeding and perforation [2,3,4]. PUD (incidence of 0.1-0.3% per year) affects about 5-10% of the worldwide population and varies according to age, sex, and geographic location [2]. This condition is frequently associated with serious complications, including heavy bleeding, perforation, gastrointestinal obstruction, and malignancy. Currently PUD has decreased significantly, but has not disappeared. The etiologies of this disease are diverse and heterogeneous and demand selective therapies to control and reduce their complications [3]. It is the most predominant gastrointestinal disease and remains a worldwide health problem due to its high morbidity, mortality, and serious therapeutic challenges [2,4,5].

PUD results from an imbalance in mucosal defensive factors, such as mucus secretion, bicarbonate efflux, endogenous antioxidant, cell regeneration, continuous synthesis and release of prostaglandin E_2_ (PGE_2_), nitric oxide (NO), and sulfhydryl compounds (SH); and aggressive agents such as smoking, alcohol consumption, dietary factors, stress, prolonged and excessive intake of nonsteroidal anti-inflammatory drugs (NSAIDs), and *Helicobacter pylori* (*H. pylori*) infection, among others [6,7,8,9]. For a long time, it was believed that the main factor implicated in the development and progression of peptic ulceration was an hypersecretory acidic environment and together with dietary factors and/or stress was thought to cause most of PUD. But the discovery of *H. pylori* infection and the widespread use of NSAIDs in the second half of the 20^th^ century changed this perception. In recent years, peptic ulcer has been found to have multiple causes—*H. pylori* infection, NSAIDs, smoking, alcohol consumption, stress, lifestyle, and genetic predispositions are determined as major risk factors for the development of PUD [2].

## 2. Pathophysiology 

Under normal conditions, gastric and duodenal mucosa integrity is maintained by the mucus-bicarbonate barrier, the neutral pH, and continuous epithelial cell renewal [10,11]. PGE_2_ stimulates cell proliferation, mucus, and bicarbonate production, promoting a crucial function in mucosa preservation. Another vital factor in gastric homeostasis is adequate blood flow. The NO and PGs are responsible for the maintenance of proper perfusion to the gastric mucosa, assuring the delivery of oxygen and nutrients, as well as removing toxic metabolites, preventing damages to the tissue [12].

The etiology of PUD is a complex and multifactorial process that can involve smoking, ingestion of alcoholic beverages, *H. pylori,* and NSAIDs. Smoking has several negative effects, including inhibition of epithelial renewal, increase of gastric acid production, and decrease of bicarbonate production [13]. Alcohol disrupts the mucosal barrier and increases its permeability; even though short-term exposure is rapidly recovered, prolonged exposure by frequent consumption of alcoholic beverages may lead to more severe injuries [14].

*H. pylori* infection is considered one of the most frequent and important causes of PUD. The discovery that *H. pylori* infection is a major cause of PUD revolutionized the views on the etiology and treatment of the disease with invaluable benefits to millions of people worldwide [15]. As the human stomach is a hostile place for most bacteria, *H. pylori* developed a mechanism of acid resistance that, together with colonization factors, help the bacteria overcome the mucosal barrier [16]. After escaping the antimicrobial gastric acid, the bacteria then enter the mucous layer and adhere to the gastric mucosa, where it triggers an inflammatory response and gastric injury [13,17].

Some evidence suggests that the elimination of *H. pylori* infection alone is sufficient to heal peptic ulcers and prevent recurrent bleeding [2]. However, the eradication of *H. pylori* infection does not completely abrogate its high morbidity and substantial mortality. It has been demonstrated that in *H. pylori*-negative patients, the incidence of the disease increased in large part due to the generalized use of low-dose NSAIDs, such as acetylsalicylic acid, supporting a multifactorial etiology of PUD [18].

The lesion of the gastroduodenal mucosa induced by NSAIDs occurs in two ways: a topical damage and a systemic lesion. The NSAIDs induce topical damage through a disturbance of the gastric epithelium, subsequent diffusion of hydrogen ions, and by altering the hydrophobic characteristics of the gastric mucosal surface, allowing gastric acid and pepsin to injure the epithelium [19]. The second way involves systemic injury induced by the inhibition of the synthesis of cyclooxygenase and endogenous PGs, which results in the decrease of epithelial mucus, bicarbonate secretion, mucosal blood flow, epithelial proliferation, and mucosal resistance to injury [13,20].

## 3. Treatment and Management of PUD

Prior to the discovery of *H. pylori*, ulcers were known to recur, and for many years the standard practice was to maintain patients on acid-suppressive drugs. Since the dictum “no acid, no ulcer” was coined, the development of medical therapies to combat PUD has targeted the secretion of gastric acid and mucosal defense mechanisms [18]. Anti-ulcer drugs such as proton pump inhibitors (PPIs), prostaglandin analogues (misoprostol), and histamine-2 receptor antagonists (H2RAs) have been developed for mucosal protection, healing of mucosal damage, and are still prescribed for prevention of PUD [21]. Nowadays, the treatment of PUD consists of healing and prevention of the complications and should include anti-ulcer drugs and appropriate management of risk factors. Patients should be advised to stop smoking and alcohol abuse, participate in stress management programs, and avoid the use of NSAIDs [18]. However, after the discovery of the *H. pylori,* the standard first-line therapy for the treatment of this bacterium consists of a PPI and two antibiotics, such as clarithromycin and amoxicillin or metronidazole administered for 7-14 days (triple therapy) or with bismuth/tetracycline (quadruple therapy) [22,23,24,25]. Nevertheless, *H. pylori*’s increasing resistance to antibiotics has dropped from more than 90% two decades ago to less than 70% at present in many countries [2,24,25]. Besides, the high resistance of *H. pylori* to clarithromycin can decrease the success rate of clarithromycin-based triple therapy by up to 70% [5,26]. One study found that treatment of patients infected with clarithromycin-resistant *H. pylori* failed almost completely [6,27]. 

Several studies have evaluated the safety and efficacy of vonoprazan, a new acid suppressant used in the treatment of acid-related disorders [28]. This novel drug competes with K^+^, preventing it from biding to the gastric H^+^/K^+^-ATPase. This drug has been clinically used in Japan for short-term treatment of PUD and *H. pylori* infection based on their effectiveness in the eradication of clarithromycin-resistant *H. pylori* strains. However, when long-term acid suppression treatment is needed, side-effects such as hypergastrinemia, pneumonia, bacterial overgrowth in the small intestine, and infection with *Clostridium difficile* may occur, even with the classic anti-ulcer drugs such as PPIs or even with vonoprazan [28,29]. A new vaccine for primary prevention against *H. pylori* is currently under development [30].

Due to the decrease in the efficacy of first-line treatments, increase of side-effects, and worldwide reports of *H. pylori*‘s resistance to antibiotics, new approaches are needed to manage this problematic infection; thus, efforts are being directed towards the development and delivery of new anti-*H. pylori* drugs [31]. Natural products, especially compounds derived from medicinal plants, have been sought as a source of new medicine, due to the great variety of chemical structures and structural modifications [32,33,34,35,36]. The essential oils produced by several medicinal plants are rich in monoterpenes, which are compounds with a great diversity of biological activities and potential therapeutic applications [37]. The class of monoterpenes that have been recognized as analgesic and anti-inflammatory properties stands out [38]. Monoterpenes also exhibit anti-ulcer, healing, and antimicrobial activities; and are a pharmacological alternative for the treatment of peptic ulcers of different etiologies, including *H. pylori* infection related [39].

## 4. Monoterpenes, Peptic Ulcers, and *H. pylori*

Monoterpenes are a class of terpenes that contain two isoprenes in their molecules [40]. They are important components of the interaction between plants and their environment [41]. These substances are considered ubiquitous since they are present as a major compound in many essential oils of vegetal species used in the food industry as additives and flavoring agents, such as *Cymbopogon citratus* (DC.) Stapf. (lemongrass), *Citrus limon* (L.) Osbeck (lemon), and *Origanum vulgare* L. (oregano) [42,43,44,45]. Recent studies have revealed their pharmacological activities, including antimicrobial, antitumor, antioxidant, analgesic, anti-inflammatory, and anti-ulcerogenic actions [46,47,48,49]. This study was carried out based on the extensive search of existing literature of the monoterpenes with anti-ulcer activity against PUD. Information of 20 monoterpenes is given in this article regarding chemical structure, in vivo or in vitro experimental models, acute or chronic effects (dose, route and vehicle used), and the mechanisms of action. To select these monoterpenes, terms such as “monoterpenes”, “peptic ulcer disease”, “gastric ulcer”, “peptic ulcer”, “gastroprotective”, or “gastric healing” were used. A search was performed in the scientific literature databases (Scopus, ScienceDirect, PubMed, Web of Science, Medline, Springer, and Google Scholar) for data up to November 2019.

Table 1 summarizes the monoterpenes that could be candidates for new anti-ulcer drugs based on in vivo and in vitro experimental models. We chose to only include studies conducted with isolated monoterpenes and exclude those in which monoterpenes were components of essential oils and other compounds to avoid the possible effects of interactions (synergisms and/or antagonisms between compounds). Several experimental models have been utilized to evaluate anti-ulcer drugs, but they are mainly used to investigate the preventive (gastroprotective) and curative (healing) properties of anti-ulcer agents. The monoterpenes presented are potential therapeutic targets for the treatment of ulcers and were selected based on their effects in animal models against the ingestion of noxious exogenous agents such as NSAIDs and ethanol or via oxidative stress (simulated by the ischemia-reperfusion process). 

Table 1 also presents the healing capacities of some important monoterpenes, including substances able to heal gastric wounds induced by acetic acid, an experimental model that simulates chronic gastric ulcers in humans. Table 1 indicates the lack of studies on the anti*-H. pylori* effects of these relevant monoterpenes, which may instigate researchers to evaluate the action of these compounds as multitarget agents. In this review, we also include, in Table 2, studies that have advanced the anti-ulcer effects of monoterpenes, including gastroprotective actions, healing effects, and/or those compounds that have antimicrobial activities against *H. pylori*. The monoterpenes were then evaluated against each of the most common aggressive agents, such as NSAID, ethanol, ischemia-reperfusion (I/R) process, acid acetic, and *H. pylori*.

## 5. NSAIDs

NSAIDs are one of the most widely used drugs in the world, causing a substantial increase in the risk of upper gastrointestinal complications [21]. These drugs are commonly used to treat pain, fever, and inflammation [71], and also for stroke prevention [72]. However, despite its positive anti-inflammatory and analgesic effects, gastric mucosal damage as a result of NSAID treatment is described as the most serious adverse reaction to this class of compounds [12,73].

NSAIDs cause gastrointestinal ulcers and complications, mainly via the inhibition of cyclooxygenase (COX), a key enzyme in the biosynthesis of PGs. COX-1 and COX-2 are two well-identified isoforms of COX [12,73]. The COX-1 isoform is constitutively expressed in most tissues, producing PGs, essential in the protection and maintenance of the stomach, stimulating the synthesis and secretion of mucus and bicarbonate, increasing blood flow, and promoting epithelial proliferation, which is primarily responsible for the upkeep of gastric mucosal integrity. COX-2 is rapidly induced, mainly as a response to inflammatory stimuli [74].

In this context, traditional NSAIDs that inhibit the action of COX-1 and COX-2 (such as indomethacin or acetylsalicylic acid) induce stomach damage and cause a marked decrease in PGE_2_ content in the gastric mucosa [74]. This effect occurs via the inhibition of the COX-1 isoform, creating a gastric environment that favors topical attack by endogenous and exogenous agents [75]. Thus, due to the prevalence and severity of gastrointestinal complications related to NSAIDs, efforts have been undertaken to prevent mucosal injury induced by these drugs.

Monoterpenes have demonstrated substantial gastroprotective action against NSAIDs in experimental models (in vivo). These include thymol [70], ascaridole [50], citral [45], eucalyptol or 1,8 cineole [51], epoxy-carvone [52], menthol [56,57], α-terpineol [61], thymoquinone [62], carvacrol [63], limonene [43], and β-myrcene [67]. Limonene, for example, was able to reduce up to 99% of gastric ulcer formation induced by indomethacin [43] (Table 2). Other monoterpenes, such as citral, prevented gastric ulcers in 76% of cases with 25 mg/kg, the lowest dose in the study [45] (Table 1). Attention should be paid to the monoterpenes thymol [70], menthol [56,57], limonene [43], and carvacrol [63], which play vital protective roles via stimulating the increase of mucus secretion and/or PGE_2_ content in the gastric mucosa. This stimulus is responsible for increasing the protective factors and maintaining gastric mucosal integrity. However, monoterpenes such as thymoquinone [62] present effective gastroprotective effects against NSAIDs acting in another way by reducing oxidative stress and increasing antioxidant defense mechanisms. An additional therapeutic purpose for monoterpenes is to improve therapeutic efficacy of NSAIDs (such as ibuprofen and acetylsalicylic acid) and retard gastrointestinal side-effects by use of prodrugs and promoieties like menthol, thymol, and eugenol that aim to have a synergistic effect as these are natural analgesics with traditional medicinal values [76].

## 6. Ethanol

In addition to NSAIDs, ethanol is one of the most irritating exogenous agents for the gastric mucosa. It is currently considered a drug of abuse that can cause a wide range of mental, social, and physical damages. Globally, alcohol consumption results in approximately 3.3 million deaths annually (or 5.9% of all deaths) and 5.1% of the global burden of disease [77].

Ethanol rapidly penetrates the gastric mucosa, causing damage to the membrane, exfoliating the cells, and leading to tissue erosion [14] via mechanisms such as the formation of reactive oxygen species (ROS) [78], a decrease in the concentrations of SH in the contents of the gastric mucosa [79], rupture of the endogenous mucus and increase in gastric acid secretion, and damage to the gastric mucosa due to hemorrhagic lesions [80], cellular apoptosis [81], induction of lipid peroxidation, and decreased levels of GSH [82]. Ethanol also induces injury to the vascular endothelium of the mucosa, causing disorders in the microcirculation and promoting ischemia, resulting in an imbalance with substantial production of free radicals at the site [83]. In experimental models involving the administration of absolute ethanol, ethanol 70%, or ethanol plus HCl (in different proportions), pretreatment with monoterpenes significantly reduced lesion areas and increased the production of mucus, SH, NO, and PGs, exhibiting an important gastroprotective effect. In animal models, carvacrol [63], citronellol [64], geraniol [67], epoxy-carvone [52], α-pinene [60], myrtenol [58], α-terpineol [61], linalyl acetate [55], menthol [56,57], nerol [59], eucalyptol [51], limonene [43,44], thymol [70], and β-myrcene [67] reduced up to 100% of the gastric lesions caused by ethanol administration (Table 1). In this review, we observed that the isomers of two bicyclic monoterpenes presented different anti-ulcer effects. The α-pinene (30 mg/kg) reduced up to 44% of the gastric lesions induced by ethanol and β-pinene (33 mg/kg) was unable to protect gastric mucosa against ethanol. The solubility of α- and β-pinene could probably result in a lower bioavailability and these different effects [84].

## 7. Ischemia-Reperfusion (I/R)

The I/R process is another important aggressor of the gastric mucosa. Exposure of the gastric mucosa to I/R induces hemorrhagic damage caused by the increased generation of ROS, microvascular dysfunction, and the adhesion of neutrophils, leading to the enhancement of tissue lipid peroxidation, which results in mucosal injury and cellular death [85,86,87]. Since ischemia is rarely preventable, most research in the field focuses on the advancement of techniques for the early detection and identification of therapeutic targets that contribute to minimizing post-ischemia damage [87]. The monoterpenes carvacrol and β-myrcene significantly decreased ulcerative lesions, protecting 38% and 86%, respectively, in an I/R model [63,67]. Interestingly, the monoterpene β-myrcene prevented the gastric damage induced by the generation of ROS, increasing antioxidant enzymes such as GPx, glutathione reductase, and total glutathione levels in the gastric mucosal tissue [67].

## 8. Acetic Acid

One of the biggest problems with PUD formation is the chronicity of the disease, characterized by repeated episodes of healing and re-exacerbation, which is a challenge for patients and doctors [88]. Ulcer healing is a well-regulated and programmed repair process, including cell proliferation, inflammation, re-epithelialization, formation of granulation tissue, angiogenesis, and interactions between various cells and the extracellular matrix, resulting in tissue remodeling and scar formation [89]. The acetic acid ulcer model in rats is the standard model for screening of new anti-ulcer drugs because it closely resembles human ulcers in terms of both pathological features and healing mechanisms [42,89]. Some monoterpenes administered orally showed a significant reduction in gastric lesions in the acetic acid-induced ulcer model. Carvacrol (91%) [42], linalool (48%) [53], eucalyptol (43%) [51], thymol (92%) [70], ascaridole (57%) [50], and geraniol (81%) [68] effectively healed the wounded gastric mucosa in relation to a control group treated with a vehicle. Animals with gastric lesions were treated with these monoterpenes for five days (geraniol) [68], seven days (ascaridole and thymol) [50,70], or 14 days (carvacrol, linalool, and eucalyptol) [52,55,68]. The monoterpenes acted via different mechanisms to promote gastric healing, including anti-secretory effects that inhibit acid secretion and accelerate ulcer healing (e.g., ascaridole) [50], reducing the release of inflammatory mediators in damaged gastric tissues (e.g., carvacrol) [42], inducing mucosal PGE2 generation that plays a relevant role in the regulation of gastric acid secretion and the maintenance of gastric mucosa integrity (e.g., carvacrol [42] and geraniol [68]), scavengers of ROS such as geraniol [68], eucalyptol [51], and linalool [53], and also promoting regeneration of the gastric cells of the mucosa such as eucalyptol [51].

The solubility of the monoterpenes is an important factor in the evaluation of their anti-ulcer effects. Generally, these compounds exhibit inadequate solubility, usually resulting in poor bioavailability and further limiting their application [54]. Most of the monoterpenes described in Table 1 and Table 2 were solubilized in Tween 80, varying in their proportion of dilution (0.1-10%). However, some monoterpenes, such as linalool and carvacrol, were solubilized in saline solution, which raises major concerns about their actual bioavailability in the body. Nanoparticles as an effective drug delivery system have become known for their advantage at solving problems related to the solubility and bioavailability of monoterpenes. For example, previous research of linalool-loaded nanostructured lipid carriers [54] or the same linalool incorporated into inclusion complex containing β-cyclodextrin revealed significantly improved anti-ulcer effects [53].

## 9. Mechanisms of Action of the Peptic Ulcers

There are several mechanisms involved in the gastroprotective and healing effects promoted by monoterpenes. Among these mechanisms, we can highlight the reduction of acid secretion, indicated by the increasing in pH; reduction of pepsin, and reduction of lipid peroxidation, as we can observe with α-pinene, ascaridole, and eucalyptol [42,43,56,60]. In addition, monoterpenes such as menthol and carvacrol, can enhance gastroprotective factors (mucus, bicarbonate efflux, and SH), and preserve the integrity of the mucosal layer by promoting cell proliferation, which is crucial to the healing of the tissue. Eucalyptol, menthol, and geraniol also increase the release of antioxidant factors and the maintenance of PGE_2_ and NO levels, which act in the upkeep of gastric microcirculation [51,59,64,68,71,75].

The main markers used to evaluate anti-inflammatory and antioxidant mechanisms are MPO, an enzyme present in neutrophils, and MDA, a final product from the reaction between ROS and polyunsaturated fatty acids from cell membranes; both can be used as indicators of inflammatory processes [45,46,67,68,69,90]. For defense against free radical damages, the cells developed different antioxidant defenses to maintain cellular homeostasis [91]. The body has enzymatic and non-enzymatic antioxidant mechanisms. The antioxidants enzymes are catalase (CAT), GPx, and SOD [92]. The non-enzymatic antioxidant system, in turn, is formed by substances such as flavonoids from diet, vitamin E, polyphenols, albumin, carotenoids, uric acid, vitamins, and GSH [92]. The activity of antioxidant enzymes has been investigated in the pathogenesis of gastric ulcer [93]. The local formation of superoxide (O_2_•) can activate SOD, which catalyzes its dismutation to H_2_O_2_. Posteriorly, the inactivation of H_2_O_2_ in H_2_O occurs through the activation of CAT or GPx enzymes [78]. GSH is a tripeptide containing cysteine found in most aerobic organisms [94]. The antioxidant properties of GSH lies in its cysteine portion, which contains a thiol group, a reducing agent that can be oxidized in a reversible fashion [95]. GSH is found in high concentrations in the gastric mucosa acting directly as a potent antioxidant and indirectly as a substrate for antioxidant enzymes [91]. The monoterpenes menthol, thymoquinone, geraniol, limonene, thymol, and β-myrcene increase activities of antioxidant enzymes GSH, SOD, and GPx [52,53,56,60,61,62,63,64].

The participation of the antioxidant mechanism has an important role in the ability to strengthen defensive factors. Among the monoterpenes, we highlight the gastroprotective effect of thymoquinone that decreases the oxidative damage of gastric mucosa by reducing lipid peroxidation, TOS (total oxidant status), and OSI (oxidative stress index) and increasing TAS (total antioxidant status) and TT (total thiol levels) [62].

Inflammation plays a prominent role in ulcer formation and healing. TNF-α, IL-6, and IL-10 are important mediators of inflammation with nuclear NF-κB activation; thus, they were used to investigate the mechanisms underlying the anti-inflammatory effect [96]. The reduction of TNF-α levels inhibits neutrophil infiltration into the gastric mucosa and the reduction of IL-6 concentration suppresses the activation of immune cells in the inflammatory site through oxidative stress [97], while IL-10, the most important anti-inflammatory and immunosuppressive cytokine [98], suppressing the production of TNF-α, which results in the reduction of inflammatory responses [96,99]. Therefore, cytokines such as TNF-α, IL-6, and IL-10 are important factors in the severity of gastric ulcers [100]. The monoterpene limonene displayed anti-inflammatory activity by decreasing TNF-α, IL-6, and IL-1β and increasing the level of IL-10 [44,62,63].

Degradation and remodeling of the extracellular matrix (ECM) is a process of major importance during gastric ulcer formation, in which matrix metalloproteinases (MMPs) are essential. Among the different subtypes of MMPs, MMP-9 is known to play an important role in gastric damage [101]. MMP-9 expression is associated with the production and release of inflammatory mediators, such as interleukin-4 (IL-4), interleukin-1β (IL-1β), and most importantly TNF-α [102,103]. Oxidative stress and ROS in gastric glands enhance the expression of MMP-9, intensifying the gastric mucosal injury [101,103]. Thymol protects against ethanol-induced gastric ulcer by downregulating the expression of MMP-9 [69,70].

Furthermore, some monoterpenes such as myrtenol and geraniol activate γ-aminobutyric acid A (GABA-A) and TRPV-1 receptors, hence increasing the release of CGRP that acts by relaxing the smooth muscle surrounding the arterioles, resulting in elevated mucosal blood flow, increased mucus, and intracellular pH on the surface of the stomach [104,105]. ATP-sensitive potassium channel opening is also among the gastroprotective mechanisms of menthol, carvacrol, and thymol, its prostaglandin-mediated activation increases blood flow in the gastric mucosa [106].

Besides the factors mentioned above, some monoterpenes have a microbicidal property, inhibiting the growth of *H. pylori*, an agent closely linked to gastric ulcer development.

## 10. Monoterpenes with Anti-*H. pylori* Effects

The eradication of *H. pylori* infection is one of the main therapeutic approaches for the treatment of gastric ulcers. Some monoterpenes with anti-ulcerogenic and gastroprotective effects also showed in vitro antibacterial effects against *H. pylori*. The bactericidal effect of monoterpenes is mainly due to the disruption of the microorganism’s lipid membrane, which increases cell permeability and leads to an inhibition of microbial metabolism. The antimicrobial effect seems to be related to the presence of a hydroxyl group in the compound structure. This biological activity increases proportionally with the number of hydroxyl groups [46]. Carvacrol had a minimal inhibitory concentration (MIC) of 40 mg/L, and geraniol (2 mg/L) inhibited 92% of *H. pylori* growth [39,68]. Limonene and β-myrcene had MICs of 75 µg/mL and 500 µg/mL, respectively [43,67]. Safranal had a MIC_50_ of 32 µg/mL [68]. Citronellol had a microbicidal effect against *H. pylori* infection, both in vitro and in vivo [64]. Bergonzeli et al. (2002) observed that the positions of the double bond in the aliphatic chain from eugenol and isoeugenol (two isomers) suggested that this conformation of the molecule is essential for passage through the *H. pylori* membrane and isoeugenol was more active against *H. pylori* than eugenol. However, several monoterpenes, despite the great healing and gastroprotective effect, do not have an anti-*H. pylori* effect (in vitro) as is the case of thymol [70].

## 11. Conclusions

This study presented the anti-ulcerogenic, healing, and antimicrobial effects of monoterpenes in experimental models related to PUD. Here, we demonstrate the therapeutic potential of this biomolecule class as a source for the development of new therapies through action in the balance between protective and aggressive factors against *H. pylori* infection. We found a substantial lack of clinical studies in contrast to the large volume of pre-clinical studies that prove that monoterpenes are viable candidates for anti-ulcerogenic drugs. Understanding the pharmacological actions and mechanism of action of monoterpenes used for PUD may provide the scientific basis for future translation in which the knowledge from preclinical research may be applied to the clinical practice of new therapies for this disease.

## Figures and Tables

**Table 1:** Monoterpenes with gastroprotective and healing effects.

Compound	Experimental Model: Treatment (Acute or Chronic) and Doses	Effect	Mechanism
Ascaridole [50] 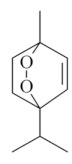	NSAID - Acute *10 mg/kg (p.o.) ~ ↓ 52% *20 mg/kg (p.o.) ~ ↓ 44%	Gastroprotective and healing effects	↓Acid secretion (↑pH) ↓ Pepsin
	**Acetic Acid (20 %) - Chronic (7 days):** 20 mg/kg (p.o.) - ↓ 57%		
	**Vehicle:** Sodium carboxymethyl cellulose		
Citral [45] 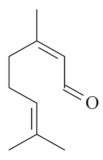	**NSAID – Acute** 25 mg/kg (p.o.) - ↓ 74.0% 100 mg/kg (p.o.) - ↓ 35.0%300 mg/kg (p.o.) - ↓ 48.0 %	Gastroprotective effect	
	**Vehicle:** Tween 80 - 1%		
Eucalyptol or 1,8-Cineole [51] 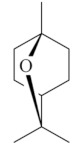	**NSAID – Acute** 50 mg/kg (p.o.) - ↓ 58.2% 100 mg/kg (p.o.) - ↓ 61.2% 200 mg/kg (p.o.) - ↓ 74.1%	Gastroprotective and healing effect	↑ Mucus (89.3%), ↑SH, ↓LPO and ↓MPO
	**Absolute Ethanol - Acute** 50 mg/kg (p.o.) - ↓ 88.1% 100 mg/kg (p.o.)- ↓ 98.5% 200 mg/kg (p.o.)- ↓ 99.2%		↑ Cell proliferation
	**Acetic Acid (30%) - Chronic (14 days)** 100 mg/kg (p.o.)- ↓ 43.1%		
	**Vehicle:** Tween 80 - 1%		
Epoxy-carvone [52] 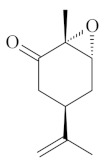	**NSAID - Acute** 10 mg/kg (p.o.) – ↓ 60.4 % 30 mg/kg (p.o.) – ↓ 47.9 % 50 mg/kg (p.o.) – ↓ 62.7 % **Absolute Ethanol - Acute** 10 mg/kg (p.o.) - ↓ 77.7% 30 mg/kg (p.o.) - ↓ 69.2% 50 mg/kg (p.o.) - ↓ 61.4% **Vehicle:** Tween 80 - 5%	Gastroprotective effect	
Linalool [53,54,55] 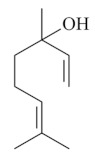	**Ethanol 90% - acute:** 33 mg/kg (p.o.) - ↓ 56.0% **Vehicle:** methylcellulose 0.1%	Gastroprotective effect	
	**Absolute Ethanol - acute:** 10 mg/kg (p.o) - ↓ 85.5% 20 mg/kg (p.o) - ↓ 76.2% 40 mg/kg (p.o) - ↓ 89.3%		
	**Acetic Acid (80%) - chronic (14 days):** 40 mg/kg (p.o) - ↓ 48.0%	Gastroprotective and healing effects	↓MPO and ↓ LPO
	**Vehicle:** Saline		
Linalyl acetate [55] 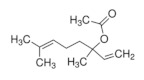	**Ethanol 90% - acute:** 36 mg/kg (p.o.) - ↓49.0% **Vehicle:** methylcellulose 0.1%	Gastroprotective effect	
Menthol [56,57] 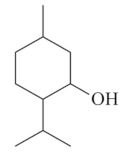	**NSAID - Acute:** 50 mg/kg (p.o) - ↓ 73.0 %	Gastroprotective effect	↓ Acid secretion ↑ Mucus and PGE_2_ ↑ Compounds SH
	**Absolute Ethanol - Acute:** 50 mg/kg (p.o) - ↓ 88.6–92.0% 100 mg/kg (p.o) - ↓ 98.4%		↑ ATP-sensitive potassium channel
	**Vehicle:** Tween 80 - 8%		↓ MPO,↑ GSH, ↑GSH-Px, ↑GR ↓ TNF-α, ↓ IL-6,↑IL-10 Anti-apoptotic effect (HSP-70, Bax)
Myrtenol [58] 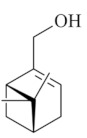	**Absolute Ethanol - Acute:** 25 mg/kg (p.o)- ↓ 40.2% 50 mg/kg (p.o)- ↓ 83.0% 100 mg/kg (p.o) - ↓ 83.2%	Gastroprotective effect	Activation of GABA-A receptors ↓ LPO
	**Vehicle:** Tween 80 - 2%		
Nerol [59] 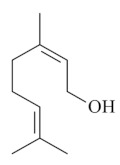	Absolute Ethanol - acute: *10 mg/kg (p.o) - ↓~94% *30 mg/kg (p.o) - ↓~82% *100 mg/kg (p.o) - ↓~92% *300 mg/kg (p.o) - ↓~94% **Vehicle:** Tween 80 - 0.5%	Gastroprotective effect	
α-pinene [60] 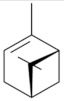	**Absolute Ethanol - Acute:** 10 mg/kg (p.o) - ↓ 48.6% 30 mg/kg (p.o) - ↓ 43.9% 100 mg/kg (p.o) - ↓ 42.1% **Vehicle:** Tween 80 - 0.1%	Gastroprotective effect	↓ Acid secretion ↑ Mucus
α-terpineol [61] 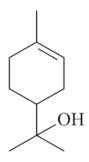	**NSAID - Acute:** 30 mg/kg (p.o)- ↓ 63.9% 50 mg/kg (p.o)- ↓ 81.3% **Ethanol 70% - Acute:** 10 mg/kg (p.o)- ↓ 66.7% 30 mg/kg (p.o)- ↓ 81.0% 50 mg/kg (p.o)- ↓ 94.1% **Vehicle:** Tween 80 - 10%	Gastroprotective effect	
Thymoquinone [62] 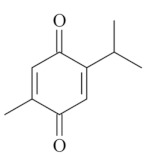	NSAID - Acute: * 20 mg/kg (p.o) - ~↓ 46%		↑ SOD, ↑GPx, ↑NO, ↓ apoptosis
	**Vehicle: **Corn oil 10%		↓iNOS,↓TOS, ↓OSI, ↓ NF-κβ, ↓ TNF-α, ↑TAS, ↑TT, ↑ADMA, ↑ DDAH-1, ↑DDAH-2

ADMA—asymmetric dimethylarginine; DDAH-1—dimethylarginine dimethylaminohydrolase-1; DDAH-2—dimethylarginine dimethylaminohydrolase-2; GPx—glutathione peroxidase; IL-10—interleukin 10; MIC—minimal inhibitory concentration; MMP-9—matrix metalloproteinase-9; MPO—myeloperoxidase; NF-κB; nuclear factor kappa B; NO—nitric oxide; NSAID—non-steroidal anti-inflammatory drugs; OSI—oxidative stress index; P.O.—administered by oral route; PGE_2_—prostaglandin E_2_; SH—sulfhydryl compounds; SOD—superoxide dismutase; TAS—total antioxidant status; TNF-α—tumor necrosis factor-α; TOS—Total oxidant status and TT—total thiol.* This data was estimated on the basis of results presented in the article.

**Table 2:** Monoterpenes with anti-*Helicobacter pylori* activity.

Compound	Experimental Model: Treatment (Acute or Chronic), Route, and Doses	Effect(s)	Mechanism(s)
Carvacrol [42,63] 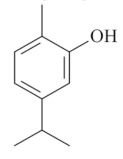	**NSAID - Acute:** 25 mg/kg (p.o) - ↓43.0% 50 mg/kg (p.o) - ↓42.0%	Gastroprotective and bactericidal effects	↑ Mucus, ↑ SH compounds,↑ NO, ↑catalase and ↑PGE_2_ level K_ATP_ channel
	**Absolute Ethanol - Acute:** 16.6 mM (p.o) - ↓48.0% 33.3 mM (p.o) - ↓41.0%		
	**Ethanol/HCl - Acute:** 8.3 mM (p.o.) - ↓ 28.0% 16.6 mM (p.o) - ↓70.0% 33.3 mM (p.o) - ↓63.0%		
	**I/R - Acute:** 16.6 mM (p.o) - ↓51.0% 33.3 mM (p.o) - ↓38.0%		
	**Acetic Acid (80%) – Chronic (14 days):** 25 mg/kg (p.o) - ↓60.0% 50 mg/kg (p.o) - ↓91.0% 100 mg/kg (p.o) - ↓81.0%		
	**Minimal Bactericidal Concentration against *H. pylori*: **0.04 g/L		
	**Vehicle:** Saline, Tween 80 -1%, saline, propylene glycol or distilled water		
Citronellol [64] 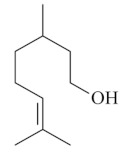	**Absolute Ethanol - Acute:** 30 mg/kg (p.o) - ↓ 72.0% 100 mg/kg (p.o) - ↓89.0% **Bactericidal activity in vivo against *H. pylori *- Chronic (7 days; 2 x day):** 12.5 mg/kg (i.p.) - ↓ 87.0% 12.5 mg/kg (p.o.) - ↓53.0% 25 mg/kg (p.o.) - ↓ 80.0% 50 mg/kg (p.o) - ↓87.0% **Vehicle:** Tween 80 - 2%	Gastroprotective and bactericidal effects	
Geraniol [65,66] 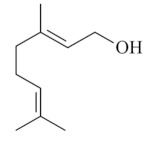	**Absolute Ethanol - Acute:** 3 mg/kg (p.o) - ↓ 55.7% 7.50 mg/kg (p.o) - ↓ 70.0%10 mg/kg (p.o) - ↓ 84.8% 200 mg/kg (p.o) - ↓ 99.0% **Acetic Acid 10% - Chronic (5 days)** 3 mg/kg (p.o) - ↓ 80.5% **Bactericidal activity in vitro against *H. pylori*:** 2 mg/L - ↓ 92.0% **Vehicle:** DMSO 10% or Tween 80 - 8%	Gastroprotective, bactericidal and healing effects	↑ Mucus (↑ mucin levels 88.5%) ↑ GSH ↓ MPO ↑ Compounds SH, NO, and PGE_2_ level ↑ CGRP and TRPV-1 activation
Limonene [43,44] 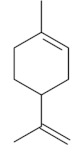	**NSAID - Acute:** 177 mg/kg (p.o) - ↓ 50.1% 245 mg/kg (p.o) - ↓ 99.0% **Absolute Ethanol - Acute:** 50 mg/kg (p.o) 100 mg/kg (p.o) 177 mg/kg (p.o) - ↓100.0% 245 mg/kg (p.o) - ↓ 99.2% **Bactericidal activity in vitro against *H. pylori*:** MIC 75 µg/mL **Vehicle:** Tween 80 - 8%	Gastroprotective and bactericidal effects	↑ Mucus Maintenance of high PGE_2_ levels ↑ GPx ↓ MPO ↓ IL-1β, IL-6 and TNF-α ↓ mRNA expression of Nf-κB, IL-1β and MPO ↑ mRNA expression of GPx
β-Myrcene [67] 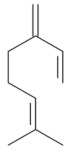	**NSAID – Acute** 7.5 mg/kg (p.o) – 74.0%		↑ Mucus ↓ MDA ↑ GPx ↑ GR
	Absolute Ethanol - Acute 7.5 mg/kg (p.o)- 60.0%	Gastroprotective effect	
	**I/R - Acute** 7.5 mg/kg (p.o) – 86.0%	Bacteriostatic effect against *H. pylori*	
	**Bactericidal activity in vitro against *H. pylori*:** MIC = 500 µg/mL		
	**Vehicle:** Tween 80 - 8%		
β-pinene [43] 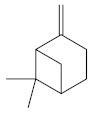	**Absolute Ethanol - Acute:** 33 mg/kg (p.o) - absent **Bactericidal activity in vitro against *H. pylori*:** MIC = 500 µg/mL **Vehicle:** Tween 80 - 8%	No gastroprotective and bactericidal effects	
Safranal [68] 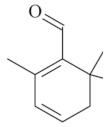	**Bactericidal activity in vitro against *H. pylori*:** MIC_50 _against *H. pylori*: 32 µg/mL **Vehicle:** not described	Bactericidal effect	
Thymol [69,70] 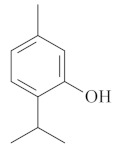	Absolute Ethanol - acute: *10 mg/kg (p.o) - ~↓ 81.0% **Vehicle:** DMSO - 5%		
	Absolute Ethanol - Acute: *10 mg/kg (p.o) - ~ 37.0% *30 mg/kg (p.o) - ~ 63.0% *100 mg/kg (p.o) - ~95.0%	Gastroprotective effect	↑SOD, ↑ GSH, ↓MPO, ↓LPO, ↓MMP-9
	**NSAID - Acute** 30 mg/kg (p.o) – 43.0% 100 mg/kg (p.o) – 49.0%	Gastroprotective effect Healing effect Bactericidal effect Absent	↑ Mucus PGE_2_ level K_ATP_ channel No involvement of NO No antisecretory action
	**Acetic Acid 30% - Chronic (7 days)** 30 mg/kg (p.o) – 91.8% 100 mg/kg (p.o) – 92.5%		
	**Bactericidal activity in vitro against *H. pylori*:** 10,000 µg/mL (absent)		
	**Vehicle:** Tween 80 - 0.2%		

CGRP—calcitonin gene-related peptide; GPx—glutathione peroxidase; GR—glutathione reductase; GSH—reduced glutathione; IL-10—interleukin 10; IL-6—interleukin 6; i.p.—administered by intraperitoneal route; LPO—lipoperoxidation; MDA—malondialdehyde; MIC—minimal inhibitory concentration; MPO—myeloperoxidase; NO—nitric oxide; NSAID—non-steroidal anti-inflammatory drugs; PGE_2_—prostaglandin E_2;_ p.o.—administered by oral route; SH—sulfhydryl compounds; SOD—superoxide dismutase; TNF-ɑ—tumor necrosis factor α; TRPV-1—transient receptor potential cation channel subfamily V member 1. * Data was estimated on the basis of the results presented in the article.
